# Dairy cattle mortality as a sensitive warning system for the effects of high and low ambient temperature on human health

**DOI:** 10.1186/2049-3258-73-S1-P23

**Published:** 2015-09-17

**Authors:** Bianca Cox, Antonio Gasparrini, Boudewijn Catry, Jaco Vangronsveld, Tim Nawrot

**Affiliations:** 1 UHasselt, Diepenbeek, Belgium; 2 London School of Hygiene & Tropical Medicine (LSHTM), London, United Kingdom; 3 Scientific Institute of Public Health (WIV-ISP), Brussels, Belgium; 4 Hasselt University, Diepenbeek, Belgium

## Background

Extreme temperatures are associated with increased mortality among humans. The use of animal sentinels may improve causality of epidemiologic findings in humans and may provide additional insights. Animal sentinels have the advantage that they are less subject to concurrent exposures, bias due to confounding, and exposure misclassification than human populations. Therefore, we investigated the effects of low and high ambient temperature on the risk of mortality among dairy cattle.

## Methods

We combined a case-crossover design with distributed lag non-linear models on 87,108 dairy cow deaths in Belgium from 2006 to 2009. We used separate quasi-Poisson models for the warm and the cold season.

## Results

During the warm season both high and low temperatures were associated with significantly increased mortality risk among dairy cattle. Heat effects were acute and were followed by a deficit in mortality three to five days after the exposure. Accounting for this harvesting effect, the estimated 11-day (lag 0-10) increase in dairy cattle mortality for a 1°C increase in mean temperature above the heat threshold (16.8°C) was 2.17% (95% CI: 0.11, 4.28). Cold effects in the warm season were delayed by five days and persisted up to 18 days. Over lag 0-25 days the estimate for a 1°C decrease below the cold threshold (13.9°C) was 4.90% (95% CI: 0.40, 9.60). We did not find evidence for temperature effects during the cold season.

## Conclusions

We showed significant increases in dairy cattle mortality associated with low and high ambient temperatures and the temporal pattern of the association is very similar to that observed in humans. Therefore, dairy cows may serve as sensitive indicators of temperature-related health risks in human populations and may provide an early warning system for public health intervention.

